# Chicken DDX60 potentiates dsRNA analog-induced IFN-β signaling through synergistic interaction with MDA5

**DOI:** 10.1128/mbio.00546-26

**Published:** 2026-09-29

**Authors:** Zheliang Liu, Dan Xu, Ruihao Yu, Yinuo Wang, Xiaxia Du, Yingjie Wang, Yan Wang, Jiajia Niu, Gang Shu, Liuting Wu, Felix Kwame Amevor, Xiaoling Zhao

**Affiliations:** 1 State Key Laboratory of Swine and Poultry Breeding Industry, College of Animal Science and Technology, Sichuan Agricultural University 719503, Chengdu, Sichuan, China; 2 Key Laboratory of Livestock and Poultry Multi-omics, Ministry of Agriculture and Rural Affairs, Sichuan Agricultural University 506176, Chengdu, Sichuan, People's Republic of China; 3 Farm Animal Genetic Resources Exploration and Innovation Key Laboratory of Sichuan Province, Sichuan Agricultural University 506176, Chengdu, Sichuan, People's Republic of China; 4 Department of Basic Veterinary Medicine, Sichuan Agricultural University 506176, Chengdu, Sichuan, China; Okayama University, Kurashiki, Okayama, Japan

**Keywords:** chicken DDX60, MDA5, IFN-β, poly I:C, dsRNA analog, innate immunity

## Abstract

Double-stranded RNA (dsRNA) viruses cause devastating economic losses to the global poultry industry by triggering severe diseases in chickens; however, the anti-dsRNA innate immune mechanism in this species remains poorly defined. Critical limitations include the lack of the mammalian retinoic acid-inducible gene I (RIG-I) receptor (a core dsRNA sensor) and the unelucidated role of DEAD/H-box helicase DDX60 (a key antiviral factor in mammals) in chicken antiviral immunity. To address these knowledge gaps, we investigated the function and mechanism of chicken DDX60 (chDDX60) in anti-dsRNA innate immunity using chicken embryo fibroblast cells (CEFs) and DF-1 cells as *in vitro* models, combined with gene overexpression/knockdown, luciferase reporter assays, co-immunoprecipitation (Co-IP), immunofluorescence, molecular docking, and stimulation with the dsRNA analog polyinosinic-polycytidylic acid (poly I:C). Our results showed that chDDX60 is highly expressed in immune-relevant tissues (small intestine and spleen), induces IFN-β and downstream antiviral genes (ISG15, MX-1, PKR) via activating IRF7, interacts with chMDA5 to synergistically enhance IFN-β activation, acts upstream of chMDA5 to trigger the chMDA5-chMAVS-chSTING-chIRF7-IFN-β signaling axis, and is essential for establishing an antiviral state against dsRNA viruses under poly I:C stimulation. Taken together, our findings identify chDDX60 as a positive regulator of dsRNA analog-induced IFN-β signaling in chicken cells and provide new insight into the regulation of RIG-I-like receptors (RLR)-mediated innate immune signaling in RIG-I-deficient avian species. Further studies using infectious RNA virus models are needed to determine whether chDDX60 contributes to antiviral defense against dsRNA and/or ssRNA viruses.

**IMPORTANCE:**

Viral RNA sensing is essential for host defense, but chickens naturally lack retinoic acid-inducible gene I (RIG-I), a major RNA sensor in mammals. This study shows that chicken DDX60 helps activate IFN-β signaling in response to the synthetic double-stranded RNA (dsRNA) analog polyinosinic-polycytidylic acid (poly I:C) by working together with melanoma differentiation-associated gene 5 (MDA5). Mechanistically, chDDX60 functions upstream of chMDA5 and promotes activation of the chMDA5-chMAVS-chSTING-chIRF7-IFN-β signaling axis. These findings help explain how chicken cells regulate RNA-triggered innate immune signaling despite the absence of RIG-I and provide insight into avian-specific antiviral immune regulation. Because this study did not use infectious RNA viruses, future studies using dsRNA and ssRNA virus models will be needed to define the antiviral function and virus-type specificity of chDDX60.

## INTRODUCTION

The innate immune system acts as a danger sensor, providing the first line of defense against invading pathogens by responding to cellular injury, infection, and dysregulated cellular processes (1, 2). When viruses invade a host, pathogen-associated molecular patterns (PAMPs) are recognized and bound with specific host pattern recognition receptors (PRRs), rapidly activating antiviral innate immunity (3). This activation triggers a signaling cascade that efficiently restricts the replication of invading pathogens and maintains homeostasis by inducing proinflammatory cytokines such as type I interferons (IFNs) and other inflammatory cytokines (4, 5).

The double-stranded RNA (dsRNA) is a major PAMP in RNA viruses, accumulating in the cytoplasm of infected cells during replication (6). The retinoic acid-inducible gene I (RIG-I)-like receptors (RLRs) are crucial cytoplasmic PRRs for recognizing intracytoplasmic viral dsRNA (7). Among them, RIG-I and melanoma differentiation-associated gene 5 (MDA5) are critical members (8). Upon binding with viral dsRNA, RIG-I/MDA5 specifically triggers mitochondrial antiviral signaling (MAVS)-dependent IFN signaling, leading to the expression of type I IFNs and inflammatory cytokines (9, 10). While the RLR pathway is generally conserved among vertebrates, chickens lack the RIG-I gene (11, 12). It is believed that chicken MDA5 (chMDA5), under stronger selection pressure, compensates for the absence of RIG-I in sensing viral dsRNA (13). A study by Cheng et al. demonstrated that the IFN-mediated innate immune responses to dsRNA viruses in chicken are synergistically regulated by successive interactions between chMDA5 and various viral nucleic acid sensors or downstream adaptor proteins, such as DEAD/H-box helicases or MAVS (13, 14). Consequently, the specific activation of the MDA5 pathway in chickens is complex and underlies their response to viral dsRNA. The smaller repertoire of innate immune genes in chickens compared to mammals, particularly for antiviral innate immunity, has directed attention to study the interaction among chMDA5 and other virus sensors or adaptor proteins to better understand the genetic mechanisms of anti-dsRNA virus innate immunity in chickens (15).

Recent studies have reported that several DEAD/H-box helicases play a role in virus recognition and responding to RLRs-mediated antiviral signaling (16–18). Notably, in our previous study, one essential IFN-inducible gene belonging to the DEAD/H-box helicases family (19), DDX60 gene, was identified as one of the most significant selective signals in the genomes of eight Guizhou local chicken breeds by whole-genome sequencing, potentially associated with rapidly evolving high disease resistance after domestication (20), suggesting that the DDX60 gene played a crucial role in chicken immunity. In mammals, the role and relative mechanisms of DDX60 in the antiviral innate immune response have been established. Ectopically expressed DDX60 protein acts as a viral nucleic acid sensor or upstream of RIG-I, suppressing RNA virus replication and promoting viral RNA degradation by activating the type I IFN signal axis (19, 21, 22). However, it remains uncertain whether DDX60 serves a similar function in antiviral innate immunity in chickens. In addition, DExD/H-box helicases are evolutionarily conserved across multiple species, including at least eight conserved motifs (18). Thus, we hypothesized that chicken DDX60 (chDDX60) plays a vital role in regulating intracellular viral RNA-triggered innate immune signaling by acting upstream of RLR (MDA5) and synergistically interacting with the MDA5 pathway, akin to mammals. This study aims to verify its functions in anti-RNA virus innate immunity and explore the underlying mechanisms.

In most studies investigating the genetic mechanisms of antiviral innate immunity related to IFN production, polyinosinic-polycytidylic acid (poly I:C) was used as a synthetic analog of viral dsRNA (23). Studies have reported that naked poly I:C primarily triggers endosomal TLR3 signaling (24), whereas cytosolic receptors, such as RIG-1 and MDA5, are specifically activated by poly I:C complexed to transfection reagents (25). Transfection of the above complex directly mimics the process by which intracytoplasmic sensors RIG-1 and MDA5 bind with viral dsRNA to induce IFN (26), due to the function of transfection reagents to enhance endosomal escape for intracytoplasmic delivery (1, 23). Therefore, poly I:C complexed with a transfection reagent was used as an intracellular dsRNA stimulation model to MDA5-IFN-β signaling-mediated anti-dsRNA virus innate responses in this study.

The present study was aimed at elucidating the role and relative mechanism of chDDX60 in dsRNA analog-induced innate immune signaling in chicken cells. *In vitro* experiment was conducted to assess the immune functions of chDDX60 and investigate its role in IFN-β-mediated responses under poly I:C stimulation. In addition, the synergistic interaction between chDDX60 and chMDA5, as well as its role and mechanism in anti-dsRNA virus function through the chMDA5-chSTING-chMAVS-chIRF7-chIFN-β signaling pathway, were systematically examined. Taken together, this study aims to verify a new key component (chDDX60) of dsRNA analog-induced RLR signaling in chickens, to enhance our understanding of the chicken innate immune system.

## MATERIALS AND METHODS

### Cell culture

Chicken embryo fibroblast cells (CEFs) were obtained from 9-day-old specific-pathogen-free (SPF) embryos following the protocol outlined in a previous study (27). Both chicken DF-1 fibroblasts (CRL-12203; ATCC, Manassas, VA, United States) and SPF embryos were sourced from the State Key Laboratory of Swine and Poultry Breeding Industry of Sichuan Agricultural University (Sichuan, China). The cells were cultured in high-glucose Dulbecco’s modified Eagle’s medium (Gibco, USA) supplemented with 10% fetal bovine serum (Gibco, Grand Island, NY, United States) and 1% penicillin-streptomycin (Gibco). The cell culture conditions were maintained at 37°C with 5% CO_2_ and 95% air-saturated humidity.

### Construction and transfection of plasmids and RNA oligonucleotides

The RNA oligonucleotides of chDDX60 (Si-DDX60-1, Si-DDX60-2, Si-DDX60-3) and chIRF-7 (Si-IRF7-1, Si-IRF7-2, Si-IRF7-3), the FLAG-tagged pcDNA3.1-chDDX60 plasmids, and the MYC-tagged pcDNA3.1-chMDA5 plasmids were designed and synthesized according to the sequences of chDDX60 (NCBI accession number: XM_004940918.5), chMDA5 (NCBI accession number: NM_001193638.2), and chIRF-7 (NCBI accession number: NM_205372.2) by Baolu BioTech Co., Ltd. (Chengdu, Sichuan, China). Moreover, the efficiency of the knockdown was assessed by examining the relative mRNA levels of specific genes in knockdown CEFs compared to the control CEFs using quantitative real-time polymerase chain reaction (qRT-PCR). The target sequences of the relative RNA oligonucleotides are shown in Table S1. Similarly, the transient overexpression efficiency of chDDX60 and chMDA5 was checked at both the mRNA and protein levels. For luciferase assays, various chicken IFN-β promoter luciferase reporter plasmids, such as pEZX-FR01-chIFN-β, pEZX-FR01-chIFN-β-IRF1, pEZX-FR01-chIFN-β-IRF2, pEZX-FR01-chIFN-β-IRF3, and pEZX-FR01-chIFN-β-NF-κB, were synthesized by Baolu BioTech Co., Ltd., containing −158 to +14, −604 to −591, −93 to −82, −75 to −63, and −62 to −52 of the chicken IFN-β promoter fragment between MluI and BamHI sites. The vectors of pEZX-FR01-chIFN-β-IRF1, pEZX-FR01-chIFN-β-IRF2, pEZX-FR01-chIFN-β-IRF3, and pEZX-FR01-chIFN-β-NF-κβ were constructed by inserting five copies of the relative binding positive regulatory domain as described previously (13, 28). The schematic diagram of the construction of the reporter gene vectors is shown in Fig. S1.

For transient overexpression or exogenous knockdown, the CEFs or DF-1 cells were transfected with RNA oligonucleotides or plasmids using Lipofectamine 3000 (lip3000, Invitrogen, Carlsbad, CA, United States) with Opti-MEM (Gibco) according to the specifications of the manufacturer.

### Detection of immunological function of chDDX60

The relative mRNA and protein expressions of IFN-β, ISG15, MX-1, PKR, MDA5, IL-1β, IL-6, and IL-8 in the lysates of pcDNA3.1-chDDX60-FLAG plasmids (2,500 ng) and optimal Si-DDX60 (50 pmoL) transfected CEFs were detected via qRT-PCR and western blot analysis. The cells transfected with empty plasmid (pcDNA3.1) and the respective nontargeting control RNA oligonucleotides (NC-SiRNA, Baolu BioTech Co., Ltd.) were considered as controls.

### Activation of innate immune response by poly I:C *in vitro*

For stimulation of innate immune response *in vitro*, the CEFs seeded in 6-well plates were transfected with poly I:C (MCE, New Jersey, United States) at doses of 0, 0.5, 1, 2, and 4 µg/uL for predetermined time points of 0, 3, 6, 9, 12, and 24 h, using Lipofectamine 3000 (Invitrogen) according to the manufacturer’s instructions. Subsequently, the supernatants and total RNA of the CEFs were collected, and the concentration of IFN-β in supernatants, as well as the relative mRNA expressions of chIFN-β in the CEFs, were determined to obtain the optimum concentration and time of poly I:C transfection.

### Establishing protein-protein interaction network

The String database (https://www.string-db.org/) was used to predict the protein-protein interaction relationship between chMDA5 and chDDX60 (29).

### Molecular docking

The core protein structure was obtained from the PDB database (http://www.rcsb.org/), HDOCK SERVER software (http://hdock.phys.hust.edu.cn/), and ClusPro software (https://cluspro.org/) were then used to link two proteins for molecular docking. The results were visualized and analyzed using Visual Studio Code software and PyMOL software 4.0.

### Co-immunoprecipitation assay

For exogenous co-immunoprecipitation (Co-IP) experiments, DF-1 cells were seeded in 60 mm dishes (1 × 10^7^ cells/dish) overnight and co-transfected with 6 µg of pcDNA3.1-chDDX60-FLAG and pcDNA3.1-chMDA5-MYC or empty pcDNA3.1 plasmids, respectively. At 72 h post-transfection, the medium was removed carefully, following precooled DEPC water washing three consecutive times. Then, the cells were lysed with 500 µL of phenylmethylsulfonyl fluoride (PMSF, Yeasen, Shanghai, China) contained in RIPA lysis buffer (Beyotime, Shanghai, China). Furthermore, the lysates were centrifuged at 14,000 rpm for 15 min to obtain the supernatant, and immediately precipitated with a mouse anti-FLAG mAb or mouse anti-MYC mAb (Zhengneng BioTech Co., Ltd., Chengdu, Sichuan, China) in conjunction with protein A-Sepharose beads (Biotool, Houston, TX). The beads were washed with precooled TBS five consecutive times and eluted with 5× SDS loading buffers (Beyotime, Shanghai, China) by boiling for 10 min. The cell lysates were also mixed with 5× SDS loading buffer and boiled. Thereafter, the proteins eluted from the beads and cell lysates were separated by SDS-PAGE and analyzed by western blotting using the related antibodies as described previously (13).

### Luciferase reporter assay

The DF-1 cells seeded in 24-well plates were co-transfected with various overexpression plasmids (pcDNA3.1-chDDX60-FLAG and pcDNA3.1-chMDA5-MYC), RNA oligonucleotides (Si-DDX60 and Si-IRF7), or empty vector with reporter plasmids (pEZX-FR01-chIFN-β, pEZX-FR01-chIFN-β-IRF1, pEZX-FR01-chIFN-β-IRF2, pEZX-FR01-chIFN-β-IRF3, and pEZX-FR01-chIFN-β-NF-κβ) at different concentrations. After 36 h, poly I:C or not was transfected into the cells; detailed treatment designs were presented in Table S2. At 48 h post-transfection, the cells were lysed using 100 μL 1× Cell Lysis buffer (Genecopoeia, Rockville, MD, United States), after which the samples were assayed for firefly and renilla luciferase activity using the Luc-Pair Duo-Luciferase HS Assay Kit (Genecopoeia). Furthermore, the activity was normalized to Renilla luciferase activity. The reporter assay was repeated at least three consecutive times.

### Immunofluorescent staining and confocal imaging

The CEFs seeded in 6-well plates were fixed in a pre-cold 10% neutral buffered formalin. After washing with cold PBS three times, the fixed cells were blocked in 0.1% Triton-X and 10% BSA in PBS for 1 h and then incubated with primary antibodies (Vimentin, FLAG, and MYC) overnight in PBS at 4°C. Later, the cells were rinsed in PBS three times and incubated in the fluorescent-labeled secondary antibody for 1 h. Thereafter, fluorescent imaging was performed using a confocal laser-scanning microscope (Zeiss LSM800 Meta; Carl Zeiss Microimaging GmbH, Göttingen, Germany) (30).

### Detection of IFN-β signaling pathway activation role of chDDX60 in anti-dsRNA innate immunity

CEFs seeded in 6-well plates were co-transfected with poly I:C, various overexpression plasmids (pcDNA3.1-chDDX60-FLAG and pcDNA3.1-chMDA5-MYC), or RNA oligonucleotides (Si-DDX60 and Si-IRF7); detailed treatment designs were presented in Table S3. Whereas, the cells transfected with empty plasmid (pcDNA 3.1) or NC-SiRNA (Baolu BioTech Co., Ltd.) were regarded as the control. Moreover, poly I:C was transfected into the cells at the last 12 h of transfection. Then, at 48 or 72 h post-transfection, the cells were harvested for subsequent mRNA or protein analysis, respectively.

### Enzyme-linked immunosorbent assay analysis

The concentrations of IFN-β in the supernatants of the cultured CEFs and DF-1 cells were determined using enzyme-linked immunosorbent assay kits (Suolaibio, Shanghai, China) following the manufacturer’s instructions.

### RNA extraction and fluorescence real-time quantification PCR

After 48 h of transfection, the CEFs or DF-1 cells were harvested and washed with precooled DEPC water three consecutive times, and promptly followed by lysing with TRIzol. Later, total RNA was extracted using a commercial kit (Takara, Beijing, China). The RNA concentration was detected using a nucleic acid protein analyzer, which had a D260/D280 reverse transcription eligible range of 1.8–2.0. Thereafter, the cDNA was synthesized using a two-step cDNA synthesis kit (Takara).

Subsequently, qRT-PCR was performed according to the procedures of TB Green Premix Ex Taq (Takara). The fold changes in the mRNA expressions of the objective genes were quantified by the 2^−ΔΔCt^ method, where ΔΔCt is the detected ΔCt-reference ΔCt and ΔCt is the Ct _target gene_ − Ct _housekeeping gene_ (31). Moreover, the *GADPH* gene was used for normalizing the expression of the aforementioned genes as an endogenous control. Primers used in the present study (Table S4) were designed by Premier 5 (PREMIER Biosoft International, New York, United States) and synthesized by Qingke BioTech Co., Ltd. (Guangzhou, China).

### Protein extraction and western blot analysis

CEFs or DF-1 cells seeded in six-well plates were harvested after 72 h transfection, followed by three washes with precooled DEPC water and lysed with RIPA lysis buffer (Beyotime, Shanghai, China). Lysates were centrifuged at 14,000 rpm for 15 min to obtain the supernatant, followed by mixing with 5× SDS loading buffer (Beyotime, Shanghai, China) and boiling for 10 min at 100°C. The isolated proteins from the lysates were separated via SDS-PAGE and analyzed via western blotting. The antibodies used were obtained from Zhengneng BioTech Co., Ltd. (Chengdu, Sichuan, China). Thereafter, images were captured using the Tanon 5200 imaging system (Tanon, Shanghai, China), as described previously (32).

### Statistical analysis

After recognition of normal distribution and homoscedasticity, all data were subjected to statistical analysis using SPSS 25.0 software (SPSS, Inc., Chicago, Illinois, USA). The unpaired Student’s *t*-test was used for comparative analysis between the two groups. One-way analysis of variance was used for the comparative analysis among the multiple groups combined with Duncan’s multiple range test. All parameters detected in this study are presented as the mean ± SEM. Significance was defined as *P* < 0.05.

## RESULTS

### Tissue-specific expression of chDDX60

**Fig 1:**
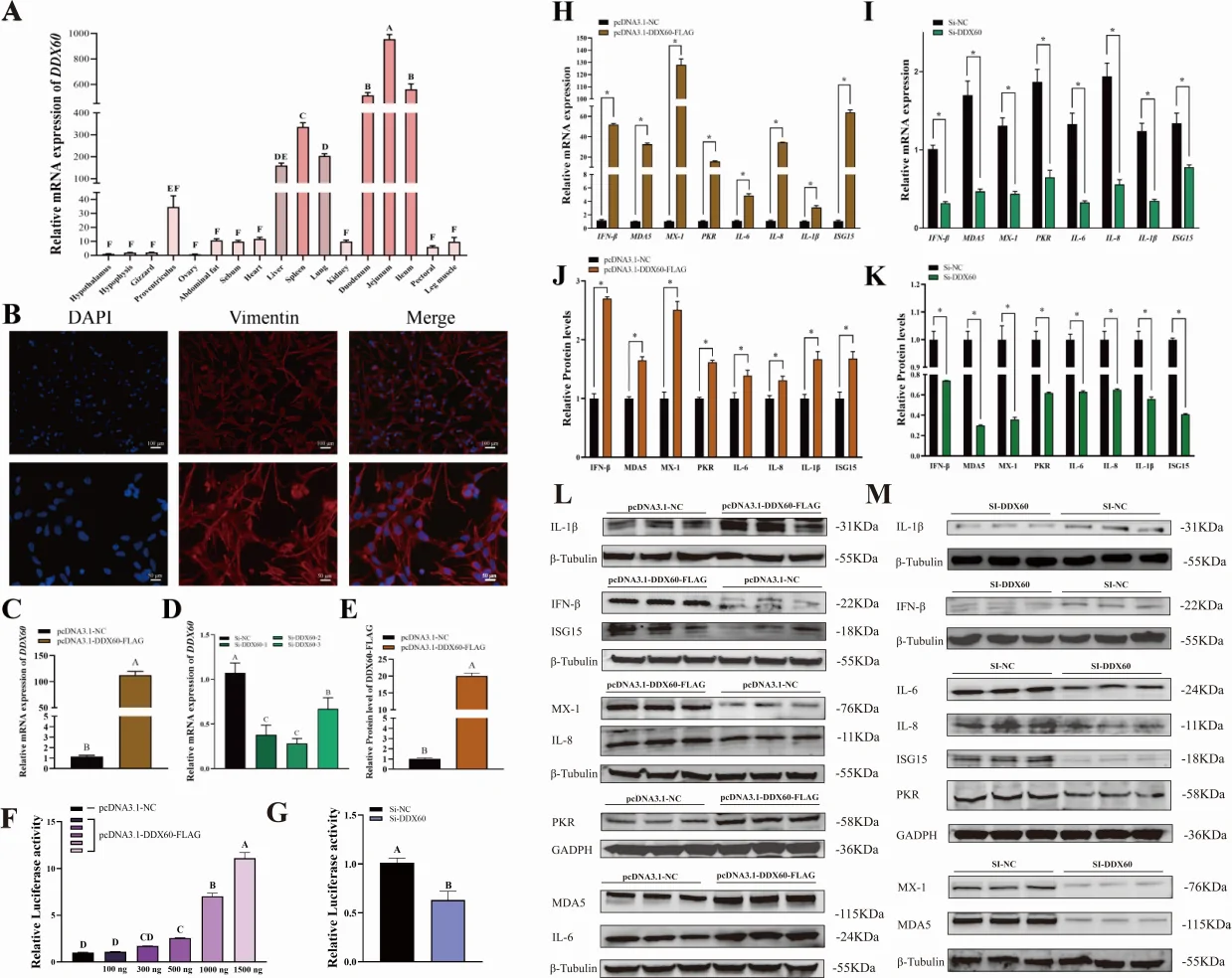
chDDX60 involved in the regulation of IFN-β, pro-inflammatory cytokines, and ISGs. (**A**) The mRNA expression of *chDDX60* in different tissues. (**B**) Immunofluorescence analysis was performed to identify chicken CEFs. Vimentin: red, CEFs marker; DAPI: blue, nucleus; Merge: identification of CEFs, 200× and 400×. (**C and D**) The mRNA expression levels of *chDDX60* in CEFs after overexpression or interference of chDDX60. (**E**) The protein expression level of chDDX60-FLAG in the CEFs after overexpression of chDDX60-FLAG. (**F and G**) The luciferase activity of IFN-β promoter in the DF-1 after overexpression or interference of chDDX60. (**H and I**) The mRNA expression levels of *IFN-*β, *MDA5*, *MX-1*, *PKR*, *IL-6*, *IL-8*, *IL-1*β, and *ISG15* in the CEFs after overexpression or interference of the chDDX60. (**J–M**) The protein expression levels of IFN-β, MDA5, MX-1, PKR, IL-6, IL-8, IL-1β, and ISG15 in the CEFs after overexpression or interference of the chDDX60. Data are presented as mean ± SEM, and * or different letters represent significance (*P* < 0.05).

chDDX60 mRNA exhibited constitutive expression across all analyzed tissues. The highest expression was observed in the small intestines (duodenum, jejunum, ileum) and spleen, which were associated with primary immunological functions. Moderate expression levels were detected in the lung and liver, while lower levels were observed in the hypothalamus, hypophysis, gizzard, proventriculus, ovary, abdominal fat, sebum, heart, kidney, pectoral, and leg muscle (Fig. 1A).

### Identification of CEFs

CEFs were harvested from 9-day-old SPF chicken embryos, and an immunofluorescence assay targeting Vimentin was conducted to characterize the CEFs (Fig. 1B), given previous findings indicating that Vimentin is a distinct protein expressed and localized within the cytoplasm of CEFs (33).

### chDDX60 involved in the regulation of IFN-β, pro-inflammatory cytokines, and ISGs

To elucidate the immunological role of chDDX60, the efficiency of overexpression or knockdown using pcDNA3.1-chDDX60-FLAG or Si-DDX60s in CEFs was initially assessed through qRT-PCR and western blot techniques. As Fig. 1 showed, the mRNA expression level of *DDX60* and the protein level of chDDX60-FLAG significantly increased compared to the pcDNA3.1 group (*P* < 0.05; Fig. 1C and E), whereas the mRNA expression of *DDX60* decreased significantly after transfection of Si-DDX60-1, Si-DDX60-2, and Si-DDX60-3 compared to Si-NC (*P* < 0.05; Fig. 1D). Among the three SiRNAs, the highest knockdown efficiency was observed in the Si-DDX60-2 group, which was renamed as Si-DDX60 and used for further experiments in this study.

To explore the potential regulatory role of chDDX60 in IFN-β production, luciferase reporter assays were conducted. The findings revealed that the overexpression of chDDX60 in DF-1 cells led to a notable dose-dependent activation of the IFN-β promoter (*P* < 0.05; Fig. 1F), whereas the significantly reduced activity of the IFN-β promoter was found after Si-DDX60 transfection (*P* < 0.05; Fig. 1G). In addition, the significantly increased mRNA and protein expression levels of endogenous IFN-β in CEFs which had been transiently transfected with chDDX60 further confirmed the IFN-β activation ability of chDDX60 (*P* < 0.05; Fig. 1H, J, and L). However, the knockdown of chDDX60 expression resulted in significantly reduced mRNA and protein expressions of IFN-β in CEFs (*P* < 0.05; Fig. 1I, K, and M).

To further understand the immune induction by chDDX60, the mRNA and protein expression levels of the RLR (MDA5), ISGs (PKR, Mx-1, and ISG15), and the pro-inflammatory cytokines (IL-1β, IL-6, and IL-8) were examined, and the results indicated that both mRNA and protein expression levels of all examined genes were significantly upregulated by chDDX60 transfection (*P* < 0.05; Fig. 1H, J, and L), whereas opposite results were observed in the Si-DDX60 group (*P* < 0.05; Fig. 1I, K, and M).

### chDDX60 regulated IFN-β production via IRF7 activation

**Fig 2:**
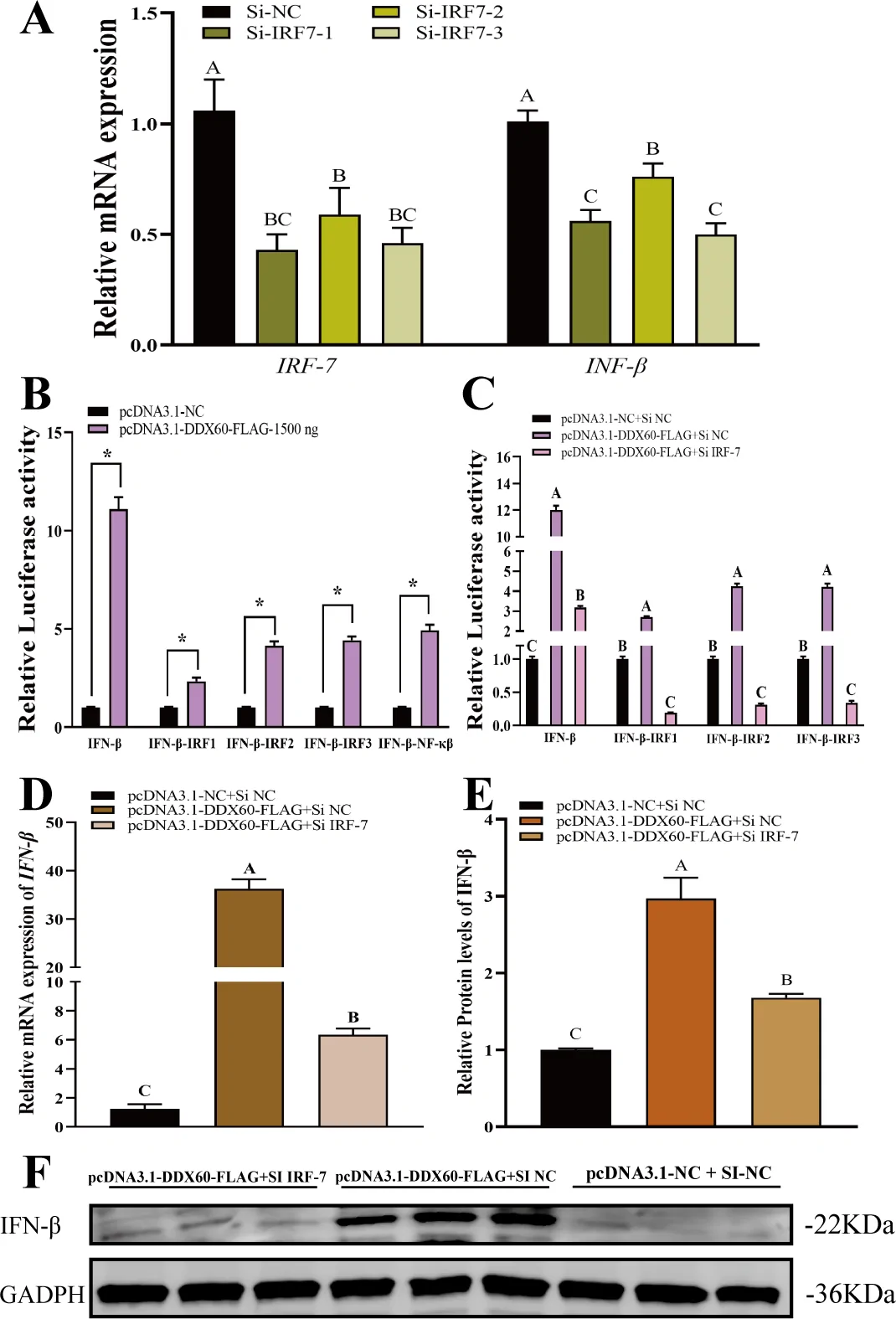
chDDX60 regulated IFN-β production via IRF7 activation. (**A**) The mRNA expression levels of *IRF7* and *IFN-*β in the CEFs after interference with IRF7. (**B**) The luciferase activity of the IFN-β promoter in the DF-1 after co-transfection of different IFN-β promoter motifs and chDDX60-FLAG. (**C**) The luciferase activity of the IFN-β promoter in the DF-1 after co-transfection of different IFN-β promoter motifs, chDDX60-FLAG, and Si-IRF7. (**D**) The mRNA levels of *IFN-*β in the CEFs after co-transfection of chDDX60-FLAG and Si-IRF7. (**E and F**) The protein expression levels of IFN-β in CEFs co-transfection of chDDX60-FLAG and Si-IRF7. Data are presented as mean ± SEM, and * or different letters represent significant difference (*P* < 0.05).

To investigate the molecular mechanism by which chDDX60 activates IFN-β, luciferase reporter plasmids (pEZX-FR01-chIFN-β, pEZX-FR01-chIFN-β-IRF1, pEZX-FR01-chIFN-β-IRF2, pEZX-FR01-chIFN-β-IRF3, pEZX-FR01-chIFN-β-NF-κB) were co-transfected with chDDX60 overexpression constructs. The luciferase reporter assay revealed significant activation of the IFN-β promoter in all treatment groups compared to the NC groups (*P* < 0.05; Fig. 2B).

To further examine if chDDX60 activates IFN-β production through IRF7 activation, the knockdown efficiency of Si-IRF7s in CEFs was first assessed. qRT-PCR results showed that the mRNA expressions of *IRF7* and *IFN-*β were significantly decreased by Si-IRF7s transfections (*P* < 0.05; Fig. 2A). Among these, Si-IRF7-3 exhibited the best knockdown efficiency, was renamed as Si-IRF7, and was used for further experiments. Subsequent luciferase reporter assays indicated that knockdown of IRF7 significantly blocked the IFN-β activation role of chDDX60 through binding to IRF domains in the IFN-β promoter motif (*P* < 0.05; Fig. 2C).

In addition, the mRNA and protein expression levels of endogenous IFN-β were analyzed after co-stimulation with pcDNA3.1-chDDX60-FLAG and Si-IRF7 to further elucidate how chDDX60 regulates IFN-β production via IRF7 activation. The results demonstrated that silencing IRF7 significantly reduced the mRNA and protein expression levels of IFN-β, even with pcDNA3.1-chDDX60-FLAG stimulation. However, these levels were still significantly higher than in the NC group (*P* < 0.05; Fig. 2D through F).

### chDDX60 synergistically interacted with chMDA5 in IFN-β activation

**Fig 3:**
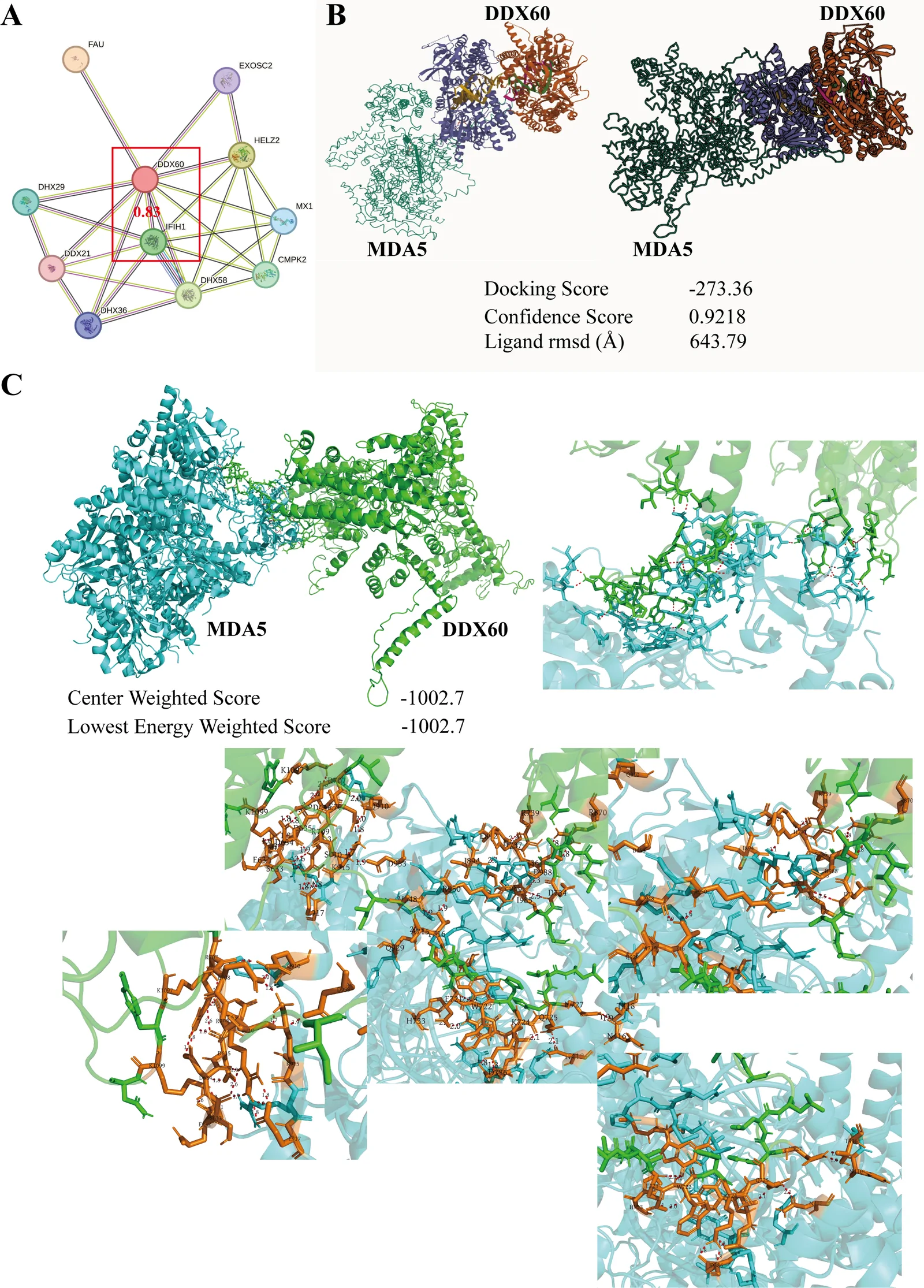
The protein-protein interaction between chDDX60 and chMDA5. (**A**) The protein-protein interaction network between chDDX60 and chMDA5 by the String database. (**B**) The molecular docking modes by HDOCK SERVER. (**C**) The molecular docking modes by ClusPro software. The amino acid in orange was binding to the loci.

The above results indicated that overexpression of chDDX60 induced both mRNA and protein expression levels of chMDA5; however, the relationship between chDDX60 and chMDA5 remained unclear. Some software works were conducted first to evaluate the protein-protein interaction between chDDX60 and chMDA5. A local clustering coefficient of 0.83 between chDDX60 and chMDA5 was observed in the protein-protein interaction network (Fig. 3A), as well as a very high docking score (binding energy of −273.36 kcal·mol^−1^ and confidence score of 0.9218) was observed between chDDX60 and chMDA5 by HDOCK (Fig. 3B). Moreover, the results of docking between chDDX60 and chMDA5 were illustrated in Fig. 3C, which showed a very high docking value (both center weighted score and lowest energy weighted score of −1,002.7) and the clear binding loci. The details of the docking between chDDX60 and chMDA5 were outlined in Table S5.

**Fig 4:**
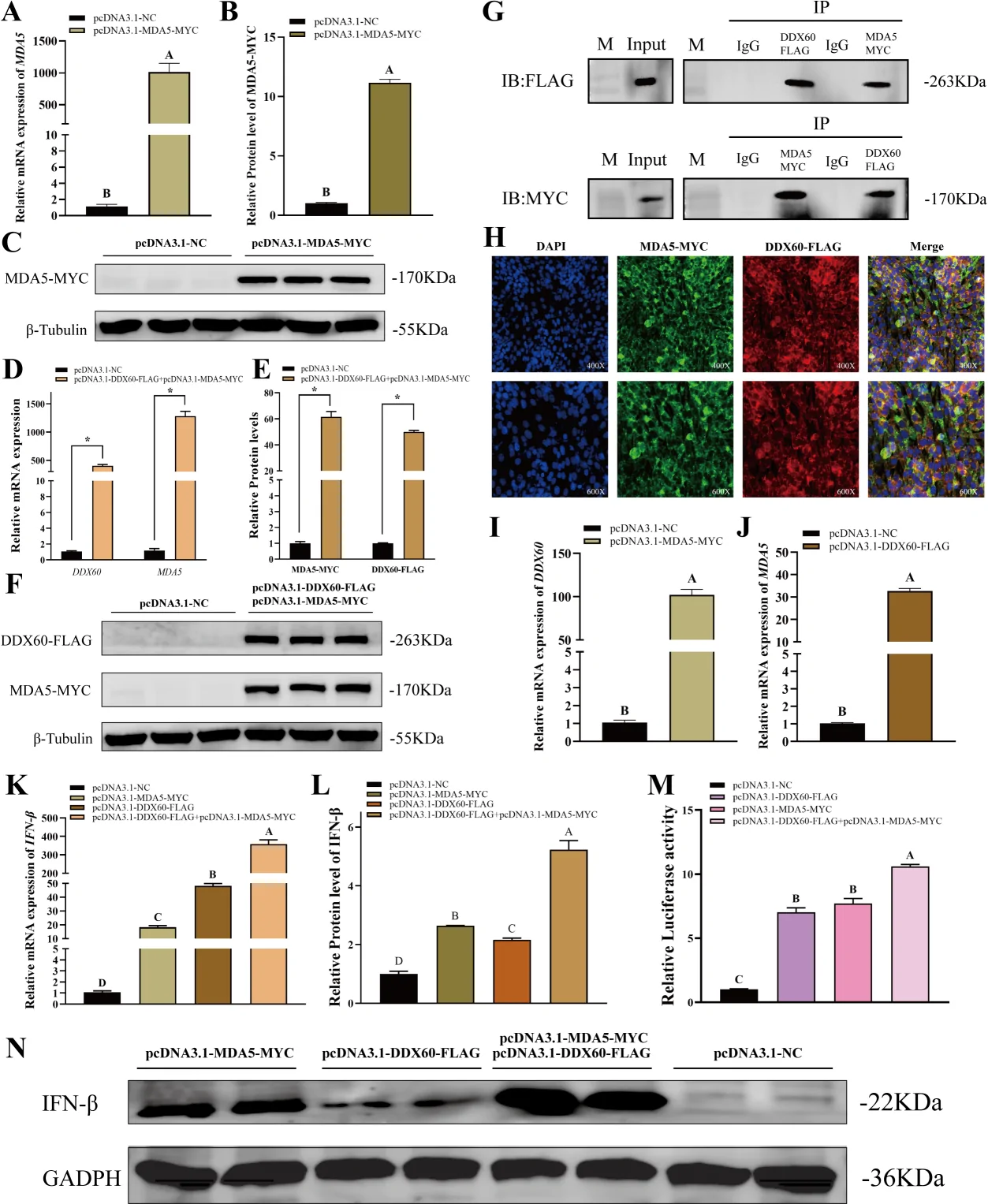
chDDX60 synergistically interacted with chMDA5 in IFN-β activation. (**A**) The mRNA expression levels of *MDA5* in the CEFs after overexpression of chMDA5. (**B and C**) The protein expression level of chMDA5-MYC in the CEFs after the overexpression of chMDA5-MYC. (**D**) The mRNA expression levels of *DDX60* and *MDA5* in the CEFs after co-transfection of chDDX60-FLAG and chMDA5-MYC. (**E and F**) The protein levels of chDDX60-FLAG and chMDA5-MYC in the CEFs after co-transfection of chDDX60-FLAG and chMDA5-MYC. (**G**) Co-immunoprecipitation assay was performed to validate the interaction between chDDX60-FLAG and chMDA5-MYC in DF-1. (**H**) Immunofluorescence analysis was performed to validate the interaction between chDDX60-FLAG and chMDA5-MYC. DAPI: blue, nucleus; MDA5-MYC: green; chDDX60-FLAG: red; Merge: identification location of chDDX60-FLAG and chMDA5-MYC in CEFs, 400× and 600×. (**I**) The mRNA expression levels of *DDX60* in the CEFs after overexpression of chMDA5. (**J**) The mRNA expression of *MDA5* in the CEFs after overexpression of chDDX60. (**K**) The expression of *IFN-*β gene in the CEFs after co-transfection of chDDX60-FLAG and chMDA5-MYC. (**L and N**) The protein expression levels of *IFN-*β in CEFs after co-transfection of chDDX60-FLAG and chMDA5-MYC. (**M**) The luciferase activities of IFN-β promoter in DF-1 after co-transfection of chDDX60-FLAG and chMDA5-MYC. Data are presented as mean ± SEM, and * or different letters represent significances (*P* < 0.05).

Further molecular biology experiments were conducted to analyze the interaction between chDDX60 and chMDA5. Initially, the overexpression efficiency of pcDNA3.1-chMDA5-MYC in CEFs was assessed. As shown by qRT-PCR and western blot results, transfection with pcDNA3.1-chMDA5-MYC significantly increased both mRNA and protein expression levels of chMDA5-MYC (*P* < 0.05; Fig. 4A through C). The interaction between chDDX60 and chMDA5 was demonstrated using a Co-IP assay and an immunofluorescence assay by co-transfecting pcDNA3.1-chMDA5-MYC and pcDNA3.1-chDDX60-FLAG or empty vectors into CEFs. And high overexpression efficiency of both chDDX60 and chMDA5 was observed in the co-overexpression system (*P* < 0.05; Fig. 4D through F). Subsequently, the Co-IP results indicated that the anti-FLAG mAb-immunoprecipitated protein complex was recognized by anti-MYC mAb (Fig. 4G). In a reverse assay, the anti-MYC mAb-immunoprecipitated complex was recognized by anti-FLAG mAb (Fig. 4G). Consistently, the immunofluorescence assay showed the same location of chDDX60 and chMDA5 in CEFs (Fig. 4H). Furthermore, the qRT-PCR results revealed that chDDX60 or chMDA5 significantly upregulated the mRNA expressions of each other (*P* < 0.05; Fig. 4I and J).

Subsequently, the synergistic role of chDDX60 and chMDA5 in signaling IFN-β response was further evaluated. The luciferase reporter assays indicated that stronger IFN-β promoter activation was induced by co-transfection of chDDX60 and chMDA5 (*P* < 0.05; Fig. 4M). At the same time, it was observed that the mRNA and protein levels of IFN-β increased significantly in the co-overexpression system, compared to each single overexpression (*P* < 0.05; Fig. 4K, L, and N).

### chDDX60 mediated IFN-β induction via chMDA5-chMAVS-chSTING-chIRF7-IFN-β signaling axis

Given that the chMDA5-chMAVS-chSTING-chIRF7-IFN-β signaling axis is a critical pathway in the IFN-β mediated antiviral innate immune response (13), and the above experiments demonstrated that chDDX60 is a chMDA5-associated protein regulating IFN-β expression by interacting with chMDA5 and activating chIRF7, further experiments were conducted to determine if chDDX60 can mediate IFN-β induction through the chMDA5 signaling pathway.

**Fig 5:**
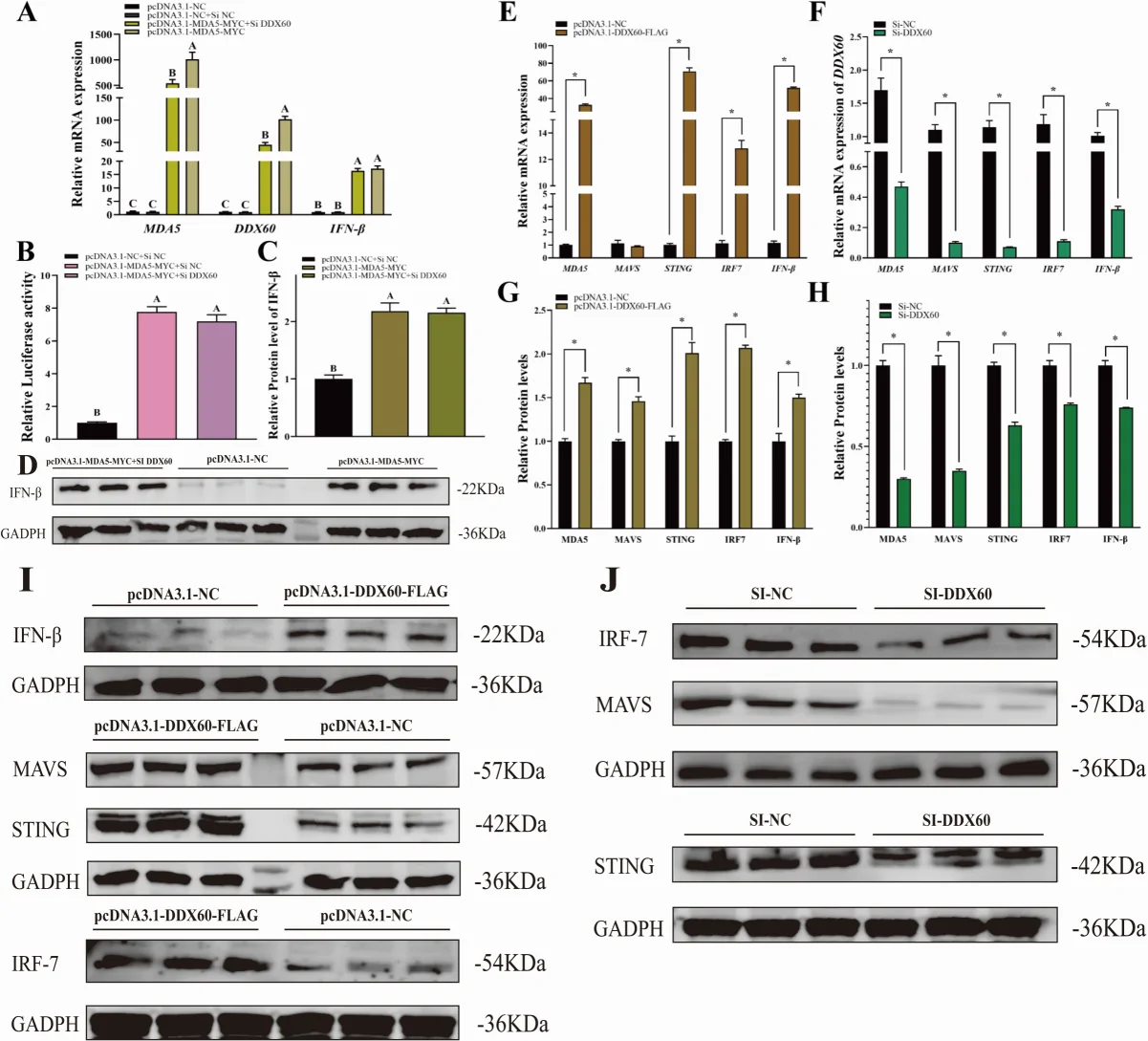
chDDX60 mediated the induction of IFN-β via the chMDA5-chMAVS-chSTING-chIRF7-IFN-β signaling axis. (**A**) The mRNA expression levels of *IFN-*β, *MDA5*, and *DDX60* in the CEFs after co-transfection of chMDA5-MYC and Si-DDX60. (**B**) The luciferase activities of the IFN-β promoter in DF-1 after co-transfection of chMDA5-MYC and Si-DDX60. (**C and D**) The protein expression levels of IFN-β in the CEFs after co-transfection of chMDA5-MYC and Si-DDX60. (**E and F**) The mRNA expression levels of chMDA5-chMAVS-chSTING-chIRF7-IFN-β signaling axis in the CEFs after overexpression or interference of chDDX60. (**G–J**) The protein expression levels of chMDA5-chMAVS-chSTING-chIRF7-IFN-β signaling axis in CEFs after overexpression or interference of chDDX60. Data are presented as mean ± SEM, and * or different letters represent significances (*P* < 0.05).

Firstly, to determine the position of chDDX60 in the chMDA5 mediated IFN-β signal pathway, the IFN-β expressions in the CEFs co-transfected with pcDNA3.1-chMDA5-MYC and Si-DDX60 were tested. The luciferase reporter assays showed that the knockdown of chDDX60 exerted no effect on the activation of the IFN-β promoter induced by chMDA5 in the CEFs (*P* > 0.05; Fig. 5B), which is consistent with both the mRNA and protein levels of IFN-β in qRT-PCR (*P* > 0.05; Fig. 5A) and western blot analysis (*P* > 0.05Fig. 5C and D;). These data indicated that chDDX60 was the upstream of chMDA5.

To further explore the role of chDDX60 in the chMDA5 mediated IFN-β signaling pathway, the mRNA and protein levels of the downstream members were assessed in chDDX60 overexpressed or knockdown CEFs, and the results showed that the mRNA expression levels of *chMDA5*, *STING*, *chIRF7*, and *IFN-*β increased significantly compared with the control group (*P* < 0.05; Fig. 5E); however, the contrast of *MAVS* levels showed no statistical significance (*P* > 0.05; Fig. 5E). Whereas, the expression levels of all the downstream proteins increased significantly after the pcDNA3.1-chDDX60-FLAG transfection (*P* < 0.05; Fig. 1L; MDA5; Fig. 5G and I). In addition, opposite results were obtained after the knockdown of chDDX60 (*P* < 0.05; Fig. 1M; MDA5 and IFN-β; Fig. 5F, H, and J). Taken together, these results confirmed that chDDX60 mediated IFN-β induction via the chMDA5-chMAVS-chSTING-chIRF7-IFN-β pathway.

### chDDX60 enhances poly I:C IFN-β production

**Fig 6:**
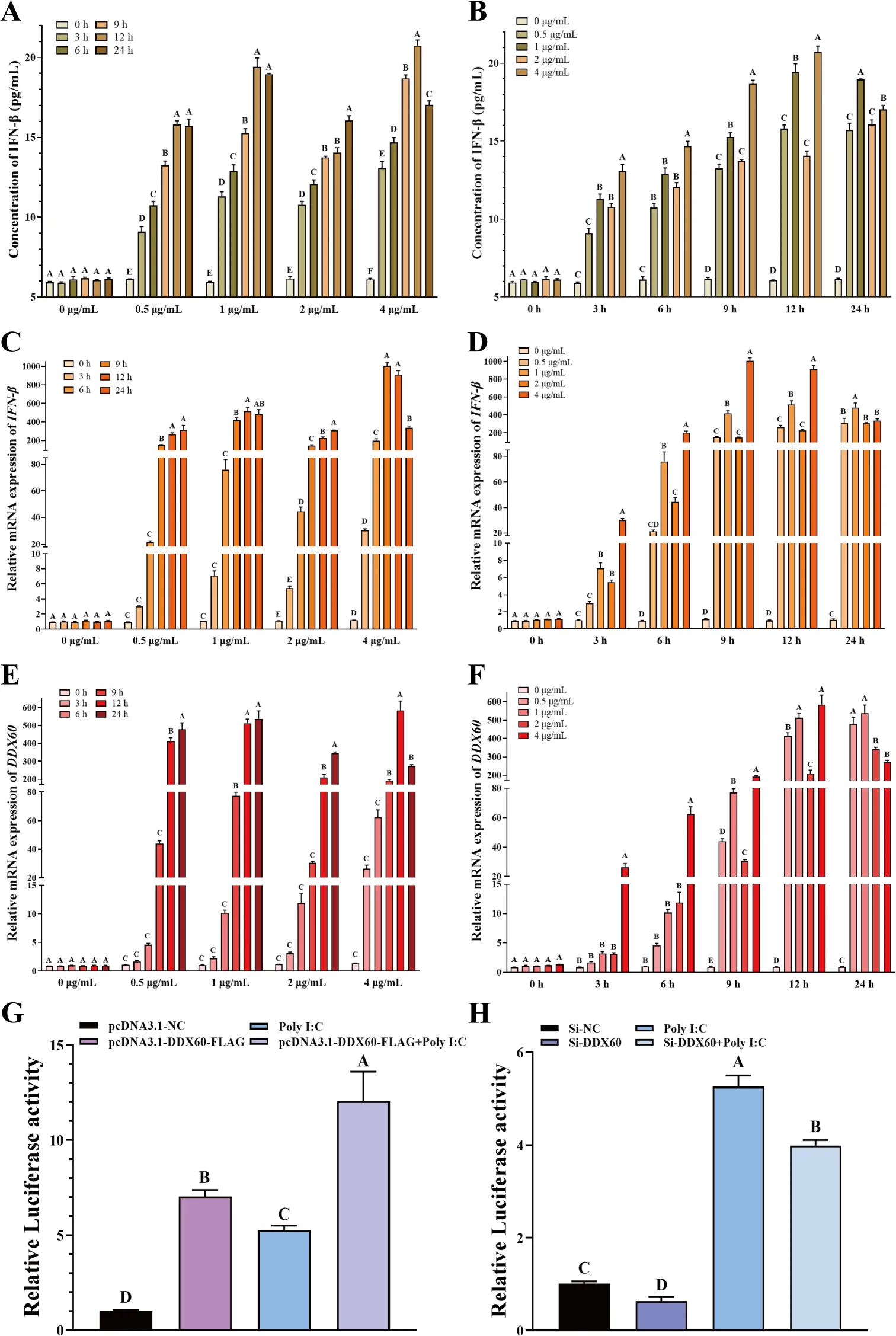
chDDX60 enhances poly I:C IFN-β production. (**A and B**) The concentrations of IFN-β in the culture supernatant of CEFs stimulated with different doses of poly I:C at different times (pg/mL). (**C and D**) The mRNA expression levels of *IFN-*β in CEFs stimulated with different doses of poly I:C for different times. (**E and F**) The mRNA expression levels of *DDX60* in CEFs stimulated with different doses of poly I:C for different times. (**G**) The luciferase activity of the IFN-β promoter in DF-1 after co-transfection of poly I:C and chDDX60-FLAG. (**H**) The luciferase activity of the IFN-β promoter in DF-1 after co-transfection of poly I:C and Si-DDX60. Data are presented as mean ± SEM, and different letters represent significant differences (*P* < 0.05).

To determine whether chDDX60 was essential for IFN-β-mediated intracellular anti-dsRNA immune response, poly I:C was used to arouse dsRNA-mediated innate immune response model in the CEFs. Firstly, the optimum transfected dose and time of poly I:C were tested. The results showed significantly higher levels of IFN-β in 12 h stimulation of poly I:C at 4 µg/uL of the concentration treatment group (*P* < 0.05; Fig. 6A through D). Further results indicated that the mRNA levels of chDDX60 increased after poly I:C transfection, and 12 h stimulation of 4 µg/µL poly I:C aroused most (*P* < 0.05; Fig. 6E and F).

Subsequent luciferase reporter assays for co-transfection of poly I:C with either pcDNA3.1-chDDX60-FLAG or Si-DDX60 revealed that IFN-β promoter activity was significantly up-regulated by pcDNA3.1-chDDX60-FLAG transfection, even within the context of a dsRNA-mediated innate immune response (*P* < 0.05; Fig. 6G). Conversely, poly I:C-stimulated IFN-β promoter activation was significantly down-regulated by Si-DDX60 (*P* < 0.05; Fig. 6H).

### chDDX60 synergizes with chMDA5 to potentiate poly I:C IFN-β signaling

**Fig 7:**
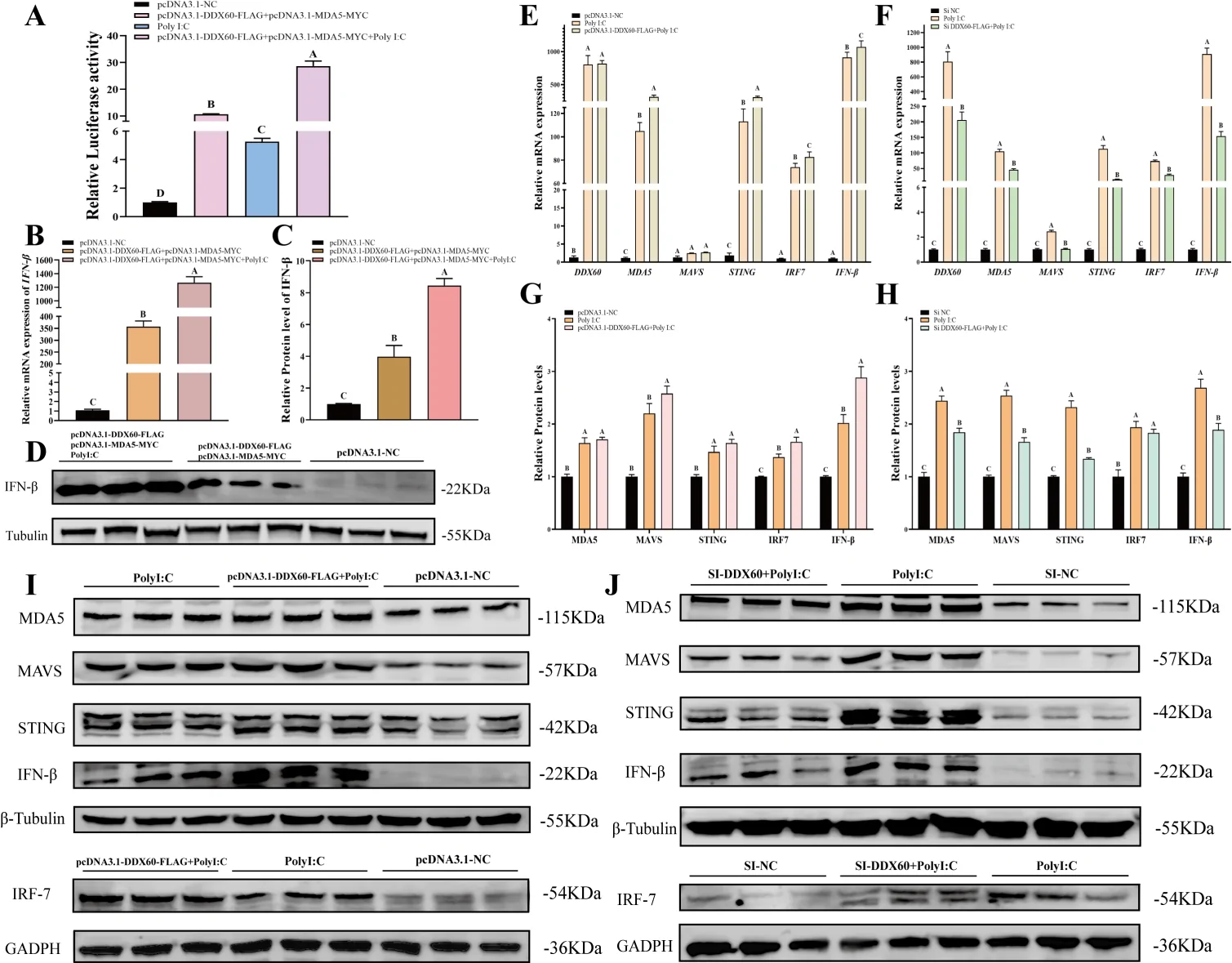
(**A**) The luciferase activity of the IFN-β promoter in DF-1 after co-transfection of poly I:C, chDDX60-FLAG, and chMDA5-MYC. (**B**) The mRNA expression levels of *IFN-*β in the CEFs after co-transfection with poly I:C, chDDX60-FLAG, and chMDA5-MYC. (**C and D**) The protein levels of IFN-β in the CEFs after co-transfection of poly I:C, chDDX60-FLAG, and chMDA5-MYC. (**E**) The mRNA levels of chDDX60-chMDA5-chMAVS-chSTING-chIRF7-IFN-β signaling axis in the CEFs after co-transfection of poly I:C and chDDX60-FLAG. (**F**) The mRNA levels of chMDA5-chMAVS-chSTING-chIRF7-IFN-β signaling axis in CEFs after co-transfection of poly I:C and Si-chDDX60. (**G and I**) The protein levels of chMDA5-chMAVS-chSTING-chIRF7-IFN-β signaling axis in CEFs after co-transfection of poly I:C and chDDX60-FLAG. (**H and J**) The protein expression levels of chMDA5-chMAVS-chSTING-chIRF7-IFN-β signaling axis in the CEFs after co-transfection of poly I:C and Si-chDDX60. Data are presented as mean ± SEM, and * or different letters represent significant differences (*P* < 0.05).

The above results indicated that chDDX60 contributes to poly I:C IFN-β production. Because chDDX60 was shown to interact with chMDA5 and to participate in chMDA5-mediated IFN-β signaling, we next investigated whether chDDX60 cooperates with chMDA5 during poly I:C intracellular dsRNA stimulation. Luciferase reporter assays, qRT-PCR, and western blot analysis showed that co-expression of chDDX60 and chMDA5 significantly enhanced IFN-β promoter activity, as well as IFN-β mRNA and protein levels in poly I:C cells (*P* < 0.05; Fig. 7A through D). Furthermore, chDDX60 overexpression increased the expression of downstream components of the chMDA5-mediated signaling axis, including chMDA5, chMAVS, chSTING, chIRF7, and IFN-β, whereas chDDX60 knockdown produced the opposite effect under poly I:C stimulation (*P* < 0.05; Fig. 7E through J). These findings suggest that chDDX60 synergizes with chMDA5 to potentiate poly I:C IFN-β signaling through the chMDA5-chMAVS-chSTING-chIRF7-IFN-β axis.

## DISCUSSION

Viral dsRNA is an important pathogen-associated molecular pattern generated during RNA virus replication and can activate innate antiviral signaling in host cells. Therefore, it is crucial to delineate the regulation of dsRNA-triggered innate immune signaling in chickens, serving as the primary defense against pathogens (34). The absence of the RIG-I gene, a critical cytoplasmic PRR for intracytoplasmic viral dsRNA recognition in mammals, has prompted investigations into the interactions among chicken dsRNA PRR (chMDA5) and other virus sensors or adaptor proteins, along with their roles in antiviral immune responses (18). While in mammals, DDX60 has been identified as a viral nucleic acid sensor or an upstream regulator of RIG-I, facilitating the suppression of RNA virus replication and promoting viral RNA degradation through the type I IFN signaling pathway (19, 21, 22). Particularly, our previous work identified the DDX60 gene as a candidate gene associated with rapidly evolving high disease resistance of eight Guizhou local chicken breeds after domestication, using whole-genome sequencing (20). However, the role of DDX60 in antiviral innate immunity in chickens remains unclear.

In the host, an effective strategy for resisting infection involves the upregulation of specific immune-related genes (18). Therefore, understanding the tissue distribution pattern of chDDX60 is crucial for refining the interpretation of its innate immune activation function. Tissue specificity is particularly important for detecting pathogens upon entry and during growth (13). Furthermore, chDDX60 mRNA was found to be highly expressed at sites of pathogen contact with immune tissue, such as the small intestine (duodenum, jejunum, ileum) and spleen. The small intestine, with its extensive mucosal area, serves as the primary barrier against invading foreign pathogens, while the spleen, being the largest lymphoid organ, suggests a functional DDX60-mediated innate immune response to pathogenic challenges in chickens.

Therefore, the activation role of chDDX60 in IFN and ISGs was assessed to further elucidate its immunological function. This study revealed that overexpression of chDDX60 led to significant upregulation of IFN and ISGs, including chicken orthologs of mammalian RLR (MDA5), ISGs (PKR, Mx-1, and ISG15), and IFN-β. Conversely, knockdown of chDDX60 hindered the ability of CEFs to induce IFN and ISGs. Furthermore, the regulation of proinflammatory cytokines in CEFs, such as IL-1β, IL-6, and IL-8, provided additional evidence for its involvement as IFN mediators (13). Consistently, previous reports have indicated that *DDX60* is an IFN-inducible gene in mammals (21), which activates type I IFN-dependent innate immunity via the RLR-mediated signaling pathway (19). However, the mechanism underlying IFN-β activation by chDDX60 remains unclear.

This study has shown that overexpression of chDDX60 resulted in the activation of the IFN-β promoter, while chDDX60 knockdown led to its reduction, consistent with findings in mammalian cells (19). Remarkably, the transcriptional regulation of IFN-β involves the coordinated assembly of an enhanceosome containing several transcription factors, including NF-κB and IRFs in chickens (13, 35). In this study, it was verified that chDDX60 activated the IFN-β promoter by binding to positive regulatory domains of IRFs or NF-κB. In mammals, reports have shown that transcription factors induce IFN-β in an IRF3-dependent manner (36, 37). However, avian species have selectively lost IRF-3 (38), with IRF7 closely resembling IRF-3 (39), suggesting IRF7 may play a pivotal role in the activation of the chicken IFN-β promoter as a transcription factor. Furthermore, specific knockdown experiments targeting IRF7 in this study confirmed its indispensability in chDDX60-mediated IFN-β activation.

Previous studies have identified chDDX60 as a critical mediator of IRF7-dependent IFN-β signaling in RIG-I-null chicken cells; however, the underlying mechanism remains unclear. Importantly, the discovery that IRF7 is an essential downstream member of the chMDA5-mediated IFN-β pathway in chickens further suggests the potential involvement of chMDA5 in the chDDX60-mediated IFN-β response (13). Several lines of evidence in this study further confirmed that chDDX60 participates in the chMDA5-mediated IFN-β response in RIG-I-free chicken cells. Firstly, the predicted results by some software indicated the interaction between chDDX60 and chMDA5 proteins, including a very high local clustering coefficient value by the String database and high docking scores by HDOCK SERVER (40) and ClusPro software (41). Moreover, the molecular docking analysis by ClusPro provided further evidence, which exhibited the 44 binding loci of chDDX60 and chMDA5 through hydrogen bonds. Subsequently, a Co-IP test and immunofluorescence analysis further revealed the interaction between chDDX60 and chMDA5. In addition, co-overexpression experiments indicated the synergistic role of chDDX60 and chMDA5 in IFN-β induction. Similarly, it has been established that various members of the DEAD/H-box helicases family, such as DDX3 (42), DDX24 (43), DHX33 (14), and DDX60 (21), interact with MDA5 in the IFN-β response in mammals. In addition, further experiments should be conducted to elucidate the specific pathway of chDDX60/chMDA5-mediated IFN-β signaling.

Indeed, it has been reported that chMDA5 can activate IFN-β by forming the chMDA5-MAVS-chSTING-IRF7-IFN-β pathway in DF-1 cells to compensate for the absence of RIG-I (13), suggesting a potential involvement of chDDX60 in this pathway to mediate the IFN-β response in chicken cells. Initially, serial overexpression and gene-specific knockdown experiments were conducted to elucidate the sequential events of the activation cascade involving chDDX60 and chMDA5. The findings demonstrated that chDDX60 functions upstream of the chMDA5 signaling pathway in chicken cells. Furthermore, the results also indicated that chDDX60 induces IFN-β by transducing the chMDA5-chMAVS-chSTING-chIRF7-IFN-β signal pathway. The observation that chMAVS protein levels were significantly upregulated by chDDX60 overexpression without corresponding changes in mRNA abundance suggests that chDDX60 enhances chMAVS stability at the post-translational level rather than promoting its transcription. chDDX60 may inhibit the degradation of chMAVS protein, thereby reinforcing the assembly and transduction efficiency of the downstream signaling complex.

In mammals, DDX60 plays a crucial role in the antiviral innate immune response by mediating the type I IFN response (44). While previous studies have identified chDDX60 as a key activator of IFN-β, whether it plays a specific role in dsRNA analog-induced IFN-β signaling in chickens remains unclear. Poly I:C, a synthetic dsRNA analog, has been reported to induce IFN-β activation through the recognition of RLRs in a concentration- and time-dependent manner (45, 46). In this study, consistent results were observed, with the highest level of activation observed at 12 h post-stimulation with 4 µg/uL poly I:C in CEFs. In addition, the expression of chDDX60 was significantly increased at 12 h post-transfection with 4 µg/uL poly I:C. Moreover, knockdown of chDDX60 markedly inhibited IFN-β production, even in response to poly I:C, while overexpression of chDDX60 upregulated it. These findings collectively suggest that chDDX60 was involved in poly I:C-induced IFN-β signaling in chicken cells. The transient increase and subsequent decrease in chDDX60 expression following poly I:C stimulation likely reflect a tight negative feedback regulation of innate immune activation. Such a pattern prevents excessive IFN-β and inflammatory cytokine production, avoiding immune overactivation and tissue damage while maintaining effective antiviral defense.

Furthermore, since chDDX60 has been identified as both a chMDA5-interacting protein and an upstream regulator of the chMDA5-mediated IFN-β signaling pathway in chicken cells, it is intriguing to investigate whether chDDX60 activates IFN-β through its interaction with the chMDA5 pathway under poly I:C-induced intracellular dsRNA stimulation. Several lines of co-overexpression tests primarily evidenced that the chDDX60 and chMDA5 synergistically activated IFN-β in poly I:C triggered innate immune response, which was the same as the previous report in mammalian cells (14). In addition, in poly I:C stimulated CEFs, the significantly increased levels of the chMDA5-chMAVS-chSTING-chIRF7-IFN-β pathway aroused by overexpression of chDDX60 demonstrated that chDDX60 acted as a central IFN-β mediator during dsRNA analog-induced innate immune signaling through the chMDA5-chSTING-MAVS-IRF7-IFN-β signal pathway. Previously, similar roles of several other members of the DEAD/H-box helicase family in chicken innate immunity have been reported, including chDDX1 (18) and chDDX3 (32). The discovery of the chDDX60-chMDA5-chMAVS-chSTING-chIRF7-IFN-β signaling axis in chickens may provide new evidence for the comparison of innate immunity characteristics between mammals and birds.

**Fig 8:**
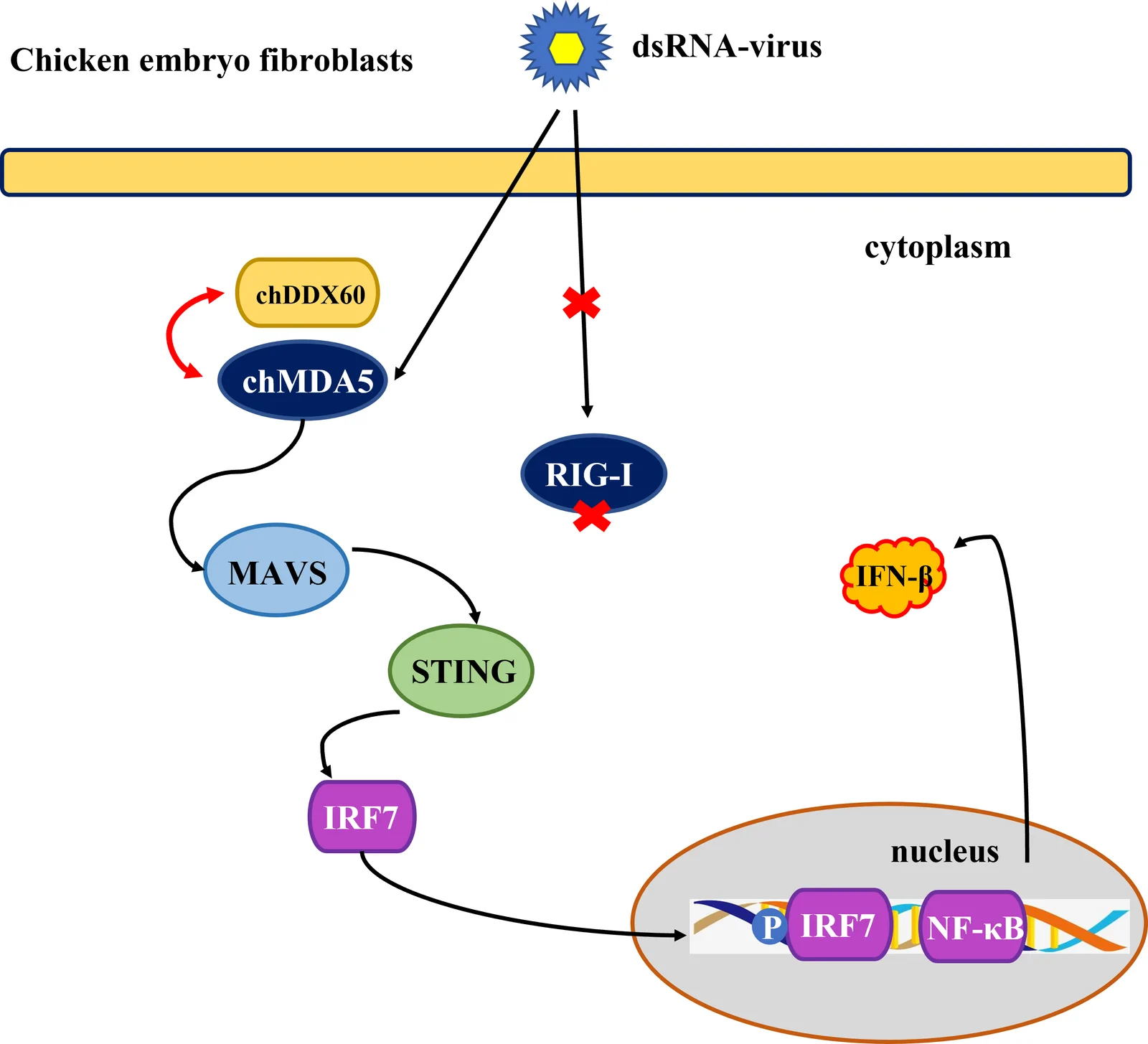
Schematic diagram of the possible mechanism of chDDX60-mediated IFN-β signaling in response to poly I:C-induced intracellular dsRNA stimulation in chickens without RIG-I. Chicken DDX60 synergistically interacts with chMDA5 under poly I:C stimulation, where it functions upstream of the chMDA5-mediated IFN-β signaling pathway, leading to activation of the transcription factor IRF7 and production of IFN-β.

Despite significant differences between the innate immune systems of chickens and mammals, the absence of certain immune genes in the chicken genome does not lead to a deficiency in the antiviral innate immune response. Compared with mammalian DDX60, which acts upstream of RIG-I, chicken DDX60 has evolved to function dependently on MDA5 due to the natural loss of RIG-I in avian species. This functional shift represents a key evolutionary adaptation that maintains robust antiviral RNA sensing in chickens. Therefore, it is essential to systematically evaluate the chicken antiviral innate immune system, which could contribute to a comprehensive understanding of the mechanisms against invading viruses in chickens. In summary, this study suggests that chDDX60 interacts synergistically with chMDA5 to induce IFN-β production in the anti-dsRNA virus immune response via the chMDA5-chMAVS-chSTING-chIRF7-IFN-β signaling axis *in vitro* (as summarized in Fig. 8). In addition, this research provides valuable reference data regarding the distinct characteristics of innate immunity in birds compared to mammals.

A limitation of the present study is that no infectious RNA viruses, including either dsRNA or ssRNA viruses, were used. Although poly I:C is widely used as a synthetic analog of viral dsRNA to activate intracellular MDA5-dependent signaling, the present data do not allow us to conclude that chDDX60-mediated antiviral activity is restricted specifically to dsRNA viruses. Given that mammalian DDX60 has been implicated in broad RNA virus-related antiviral pathways, it remains possible that chDDX60 may also contribute to innate immune responses against ssRNA viruses in chickens. Future studies using representative infectious dsRNA and ssRNA virus models will be required to determine the virus-type specificity and antiviral function of chDDX60.

### Conclusion

In conclusion, chDDX60 was identified as a critical mediator of IFN-β signaling in chicken cells. This study demonstrates that chDDX60 responds to the dsRNA analog poly I:C and promotes IFN-β induction through synergistic interaction with chMDA5, thereby activating the chMDA5-chMAVS-chSTING-chIRF7-IFN-β signaling pathway. These findings provide evidence for a distinct dsRNA analog-triggered RLR signaling mechanism in RIG-I-deficient chicken cells. Further studies using infectious RNA virus models are needed to determine whether chDDX60 contributes to antiviral defense against dsRNA and/or ssRNA viruses.

## SUPPLEMENTAL MATERIAL

Figure S1 mbio.00546-26-s0001.tiff Chicken IFN-β promoter deletion constructs in pEZX-FR01.

Supplemental legends mbio.00546-26-s0002.docx Supplemental figure and table legends.

Supplemental tables mbio.00546-26-s0003.xls Tables S1 to S5.

ASM does not own the copyrights to Supplemental Material that may be linked to, or accessed through, an article. The authors have granted ASM a non-exclusive, world-wide license to publish the Supplemental Material files. Please contact the corresponding author directly for reuse.
