# Supplementary material for: Chicken DDX60 potentiates dsRNA analog-induced IFN-β signaling through synergistic interaction with MDA5
Source: mBio. 2026 Sep 29;17(11):e00546-26. doi: 10.1128/mbio.00546-26 (PMC13753806; doi:10.1128/mbio.00546-26)
Supplement: Supplementary file 1 [file mbio.00546-26-s0002.docx]

**Supplementary Figure S1** The diagrams illustrate the pEZX-FR01 backbone constructs containing sequentially truncated fragments of the chicken IFN-β promoter, spanning from positions -158 to +14, -604 to -591, -93 to -82, -75 to -63, and -62 to -52 relative to the transcription start site. Each plasmid map displays key elements including restriction enzyme sites, the minimal promoter, reporter gene, and regulatory sequences, as indicated.

**Table S1** Sequences of siRNA oligonucleotides targeting chicken DDX60 and IRF7 genes used in this study. The GenBank accession numbers for the target genes are provided.

**Table S2** Treatment conditions and experimental purposes for each group in the dual-luciferase reporter assay. The table lists the overexpression plasmids, siRNA oligonucleotides, additional treatments, reporter constructs, and the research objective of each experiment.

**Table S3** Experimental treatment conditions for evaluating the role of chDDX60 in the activation of the IFN-β signaling pathway during the anti-dsRNA innate immune response. The table includes overexpression plasmids, siRNA oligonucleotides, and PolyI:C stimulation, along with the purpose of each treatment group.

**Table S4** Primer sequences (5′-3′) used for quantitative real-time PCR (qRT-PCR) analysis of gene expression in this study. The GenBank accession numbers for each target gene are provided. F, forward primer; R, reverse primer.

**Table S5** Hydrogen bond interactions between chDDX60 and chMDA5 identified by molecular docking analysis. The table lists the interacting amino acid residues in chDDX60 and chMDA5, as well as the corresponding hydrogen bond distances (Å).
